# Salinity Stress Tolerance in Plants

**DOI:** 10.3390/plants12203520

**Published:** 2023-10-10

**Authors:** Libia Iris Trejo-Téllez

**Affiliations:** Laboratory of Plant Nutrition, Department of Soil Science, College of Postgraduates in Agricultural Sciences, Campus Montecillo, Montecillo, Texcoco 56264, State of Mexico, Mexico; tlibia@colpos.mx

**Keywords:** salt stress, osmotic stress, ionic stress, nutrient imbalance, tolerance mechanisms, biostimulation

## Abstract

Soil salinization negatively impacts plant development and induces land degradation, thus affecting biodiversity, water quality, crop production, farmers’ well-being, and the economic situation in the affected region. Plant germination, growth, and productivity are vital processes impaired by salinity stress; thus, it is considered a serious threat to agriculture. The extent to which a plant is affected by salinity depends mainly on the species, but other factors, including soil attributes, water, and climatic conditions, also affect a plant’s ability to tolerate salinity stress. Unfortunately, this phenomenon is expected to be exacerbated further by climate change. Consequently, studies on salt stress tolerance in plants represent an important theme for the present Special Issue of *Plants*. The present Special Issue contains 14 original contributions that have documented novel discoveries regarding induced or natural variations in plant genotypes to cope with salt stress, including molecular biology, biochemistry, physiology, genetics, cell biology, modern omics, and bioinformatic approaches. This Special Issue also includes the impact of biostimulants on the biochemical, physiological, and molecular mechanisms of plants to deal with salt stress and on the effects of salinity on plant nutrient status. We expect that readers and academia will benefit from all the articles included in this Special Issue.

## 1. Introduction

The aim of this Special Issue, entitled “Salinity Stress Tolerance in Plants”, was to expand our knowledge about novel approaches dealing with the development of tolerance strategies in plants to cope with salt stress. This Special Issue contains 14 scientific articles (all original research articles) that contribute to a better understanding of the processes that can be induced in plants exposed to different saline environments. Interestingly, authors contributing to this Special Issue have affiliations with institutions in different countries, including Argentina, Canada, China, Egypt, Greece, Italy, South Korea, Mexico, Slovenia, Saudi Arabia, and Tunisia. This Foreword was written to summarize the valuable papers published in this Special Issue and to lead to the wider visibility and citation of these studies.

## 2. Salt Stress and Tolerance Mechanisms in Plants

According to global-scale studies, salt-affected soils cover an estimated global area of 831–1173 million hectares [1]. Salt-affected soils include saline, sodic, and saline–sodic soils and many sub-categories depending on the type of salt [2]. These soils contain an excessive amount of soluble salts that primarily induce ionic and osmotic stress, as well as oxidative stress and nutrient imbalances that result in various molecular, biochemical, metabolic, and physiological perturbations in plants [3]. Importantly, the salinization and sodification of agricultural land are increasing due to the impact of climate change, water shortages, and unsustainable farming practices. In particular, climate change exacerbates the accumulation of salts in soils due to dryland expansion, water scarcity, and a rise in sea levels, causing saltwater intrusion in coastal areas. This in turn leads to a rapid decline in soil health, losing its capacity for biomass production, natural filtration, carbon sequestration, and other necessary ecosystem functions [2], which drastically affects water and food security.

Different strategies have been adopted to cope with agricultural production in saline and saline–sodic soils, including leaching salt from the root zone, changing farm management practices, using biostimulation approaches, and developing salt-tolerant crops [4]. In the following paragraphs, I present a summary of the main findings reported by different research groups in this Special Issue.

El-Taher et al. [5] found that the application of salicylic acid (SA) increases the salt stress tolerance of cowpea (*Vigna unguiculata* L.) plants by improving their morphological and physiological attributes.

Zamljen et al. [6] confirmed that, based on the results of several metabolite groups, the reaction and response of metabolic content to salt stress within the *Capsicum* genus is genotype-, fruit-part-, and salinity level-dependent. 

Regarding inorganic biostimulation, Carbajal-Vázquez et al. [7] concluded that titanium (Ti) has a positive effect on the antioxidant activity and nutrition of tomato (*Solanum lycopersicum* L.) seedlings under saline stress conditions. 

Liu et al. [8] reported that nitric oxide (NO) improves the salt tolerance of the medicinal plant *Cyclocarya paliurus* (Batalin) Iljinsk by regulating the endogenous glutathione level and antioxidant capacity. 

Li et al. [9] found that salt-tolerant potato (*Solanum tuberosum* L.) can regulate physiological substances to adapt to salt stress, and ion-transport-related genes and transcription factors play a role in improving salt tolerance. 

In taro (*Colocasia esculenta* L. Schott), Baltazar-Bernal et al. [10] reported that the early application of arbuscular mycorrhizal fungi (AMF) to taro plantlets obtained in vitro is an alternative to increase or maintain the productivity of this crop in saline soils. 

In pea (*Pisum sativum* L.), a plant biostimulant obtained from *Ascophyllum nodosum* was found to potentiate the plant-growth-promotion and stress-protection activity of *Pseudomonas protegens* CHA0 under saline conditions [11].

In an approach used to evaluate the separate effects of sodium on the germination, early seedling viability, and biomass production of romaine lettuce (*Lactuca sativa* L.), tomato, beet (*Beta vulgaris* L.), and radish (*Raphanus raphanistrum* L.) in saline–sodic and alkaline forms at different concentrations, Hitti et al. [12] reported that the electrical conductivity values were higher for the saline–sodic solutions than the alkaline solutions, yielding greater fresh mass per plant for all species, with the exception of beets grown in alkaline solution, with a value of 24 mM Na^+^. The fresh mass of romaine lettuce grown in 24 mM Na^+^ saline–sodic solution was significantly greater than that of romaine lettuce grown in the alkaline solution with the same sodium concentration.

Farooq et al. [13] evaluated the amino acid profiles of rice genotypes in response to three types of salt (NaCl, CaCl_2_, and MgCl_2_) and observed that cultivars with the same origin responded similarly to each other under salinity stress conditions, whereas the amino acid profile of each rice cultivar might have depended on the origin, immune level, and genetic makeup of the respective cultivar.

Plant-associated rhizobacterial volatile organic compounds (VOCs) play a pivotal role in modulating plant salt tolerance and reducing fungal growth, suggesting that biological resources represent novel tools for counteracting the deleterious effects of salt stress and have the potential to be exploited in sustainable agriculture [14].

The Salt Tolerance-Related Protein (STRP) performs its protective functions by reducing the oxidative burst induced by salt stress, and it plays a role in the osmotic adjustment mechanisms required to preserve cellular homeostasis. Consequently, the STRP is a critical component of the response mechanisms to saline stress in *Arabidopsis thaliana* [15].

In a study aimed at deciphering the physiological responses of two olive (*Olea europaea* L.) cultivars to salt stress, Boussadia et al. [16] reported that the cultivar ‘Koroneiki’ is more susceptible to salt stress than the cultivar ‘Chemlali’, because the accumulation of glutathione peroxidase (GPX) and the decrease in catalase (CAT) and ascorbate peroxidase (APX) were more pronounced in the former cultivar.

Liu et al. [17] reported that the tomato glutaredoxin (LeGRXS14) plays a significant role in plant tolerance to salt, acting as a negative regulator in this process by exacerbating Na^+^ toxicity and the resulting oxidative stress.

Finally, DiCara and Gedan [18] concluded that by measuring species-specific responses to stress exposure, it was possible to visualize the independent and interactive effects of two components of a salinity stress regime, intensity, and duration, to reveal how species’ responses vary in magnitude and by tolerance class.

## 3. Conclusions

The research studies included in this Special Issue describe different protocols implemented to explore diverse tolerance mechanisms employed by plants to cope with salt stress. The authors reported results regarding genetic variation in salt tolerance in different plant species. They also explored the efficiency of salicylic acid, titanium, nitric oxide, arbuscular mycorrhizal fungi, seaweed, beneficial bacteria, and botanical extracts in inducing effective tolerance mechanisms in salt-stressed plants. These studies shed light on how such substances, compounds, and microorganisms may mediate or trigger tolerance responses in plants growing in saline environments. Finally, a couple of studies demonstrated the importance of the *STRP* and *GRXS* genes and their protein products in the regulation of salt stress responses in plants.

Evidently, the research articles that are published in our Special Issue are of great importance, with most of them already being cited only a few months after publication, showing the quality of the studies as well as the interest in the studies by readers and scientists.

This digital Special Issue will make this topic more accessible to a broader range of readers. It has been a great pleasure to write this foreword for such a thought-provoking, multi-authored, international publication on a unique subject. 

I express my deepest gratitude to all the contributors for their outstanding efforts in making this issue a success.
